# Berry shrivel in grapevine: a review considering multiple approaches

**DOI:** 10.1093/jxb/erae001

**Published:** 2024-01-04

**Authors:** Michaela Griesser, Stefania Savoi, Bhaskar Bondada, Astrid Forneck, Markus Keller

**Affiliations:** Institute of Viticulture and Pomology, Department of Crop Sciences, University of Natural Resources and Life Sciences, Vienna, Konrad Lorenz Strasse 24, 3430 Tulln, Austria; Department of Agricultural, Forest and Food Sciences, University of Turin, Largo Braccini 2, 10095 Grugliasco, Italy; Department of Viticulture and Enology, Washington State University Tri-Cities, Richland, WA 99354, USA; Institute of Viticulture and Pomology, Department of Crop Sciences, University of Natural Resources and Life Sciences, Vienna, Konrad Lorenz Strasse 24, 3430 Tulln, Austria; Department of Viticulture and Enology, Irrigated Agriculture Research and Extension Center, Washington State University, Prosser, WA 99350, USA; The James Hutton Institute, UK

**Keywords:** Fruit physiology, grape berry ripening, mesocarp cell death, sugar accumulation, transcriptomics

## Abstract

Grapevine berry shrivel, a ripening disorder, causes significant economic losses in the worldwide wine and table grape industries. An early interruption in ripening leads to this disorder, resulting in shriveling and reduced sugar accumulation affecting yield and fruit quality. Loss of sink strength associated with berry mesocarp cell death is an early symptom of this disorder; however, potential internal or external triggers are yet to be explored. No pathogens have been identified that might cause the ripening syndrome. Understanding the underlying causes and mechanisms contributing to berry shrivel is crucial for developing effective mitigation strategies and finding solutions for other ripening disorders associated with climacteric and non-climacteric fruits. This review discusses alterations in the fruit ripening mechanism induced by berry shrivel disorder, focusing primarily on sugar transport and metabolism, cell wall modification and cell death, and changes in the phytohormone profile. The essential open questions are highlighted and analyzed, thus identifying the critical knowledge gaps and key challenges for future research.

## Teaser

Poor sugar accumulation and mesocarp cell death in grape berries are the crucial markers of the berry shrivel ripening disorder, with hypothetical causes being found in sugar metabolism or ripening regulation.

## Introduction

Since their domestication, perhaps as early as 11 000 years ago, grapevines (*Vitis* ssp.) have remained one of the world’s most culturally and economically important fruit crops (Dong *et al.*, 2023). Grape berry development has been studied in the pre- and post-omics era to enhance our understanding of berry growth and ripening, and regulatory mechanisms (Coombe and McCarthy, 2000; Deluc *et al.*, 2007; Fortes *et al.*, 2015; Castellarin *et al.*, 2016; Fasoli *et al.*, 2018). For instance, we have accumulated extensive knowledge concerning the involvement of primary metabolism as a source of energy and precursors for downstream processes, and their contribution to organoleptic properties (Davies and Robinson, 1996; Conde *et al.*, 2007; Hayes *et al.*, 2007; Shahood *et al.*, 2020; Savoi *et al.*, 2021). Furthermore, the plasticity of secondary metabolism, including taste and aroma compounds (Castellarin *et al.*, 2011; Blancquaert *et al.*, 2019; Lin *et al.*, 2019; Cataldo *et al.*, 2021), and the responses to biotic and abiotic stresses have been well characterized (Savoi *et al.*, 2017; Cramer *et al.*, 2020; Lecourieux *et al.*, 2020; Pimentel *et al.*, 2021; Rienth *et al.*, 2021; Hewitt *et al.*, 2023).

Fruits of many species can be subject to ripening disorders, resulting in severe losses worldwide in yield and fruit quality. Despite extensive studies, the underlying physiological processes in grapevine, where ripening disorders occur in diverse forms, remain elusive (Bondada and Keller, 2012b). These disorders or syndromes include bunch stem necrosis (BSN), late season dehydration (LSD), berry splitting, sunburn, and berry shrivel (BS), also known as sugar accumulation disorder (SAD) or suppression of uniform ripening (SOUR) shrivel. The first described ripening disorders in grapevine were arguably BSN in Switzerland (Osterwalder, 1937) and LSD in California (Rosa and Nielson, 1956). Also, BS-like symptoms on wine grapes were reported in Austria in the 1980s (Stellwaag-Kittler, 1983), similar to the earlier reports with table grapes in California (Jensen, 1970). Since then, research studies have focused in more depth on the topic in order to identify the cause of BSN (Christensen and Boggero, 1985; Keller and Koblet, 1995; Hughes *et al.,* 2008), LSD (Rogiers *et al.*, 2006; Fuentes *et al.*, 2010), BS (Krasnow *et al.*, 2009; Hall *et al.*, 2010; Knoll *et al.*, 2010; Griesser *et al.*, 2012), berry splitting (Chang *et al.*, 2019; Chang and Keller, 2021), and sunburn (Rustioni *et al.*, 2014; Müller *et al.*, 2023). The commonality of external symptoms (predominantly shriveling) and the simultaneous occurrence of different disorders in vineyards have hampered progress and led to inconsistent reporting in publications.

This review focuses on advancements in knowledge of the grape ripening disorder BS, characterized by cessation of sugar accumulation shortly after the onset of ripening and subsequent berry shrinkage. As a starting point, we summarize the significant steps involved in grape berry ripening to explain the common and distinct symptoms of the economically most relevant ripening disorders. We then identify putative causal factors that might be involved in triggering the BS anomaly, and finally provide multiple insights into the most relevant open questions and challenges for future research.

## Grape ripening

**Fig. 1.**
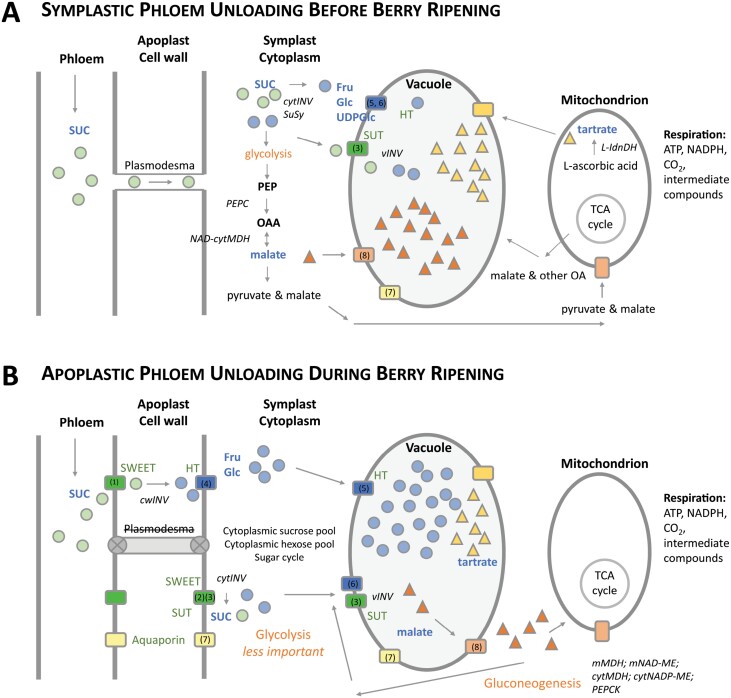
Summary of phloem unloading and sugar metabolism in grape berries. (A) Symplastic phloem unloading and glycolysis in berries before ripening onset. (B) Apoplastic phloem unloading with closed plasmodesmata after ripening onset, and malate metabolism, gluconeogenesis, and sucrose cycle in the cytoplasm. Illustration prepared with information from Dai *et al.* (2010), Kuhn *et al.* (2014), Li *et al.* (2021), Savoi *et al.* (2021), and Walker *et al.* (2021b). SUC (sucrose), Fru (fructose), Glc (glucose), PEP (phosphoenolpyruvate), OAA (oxalacetate), vINV (vacuolar invertase), cytINV (cytosolic invertase), cwINV (cell wall invertase), PEPC (cytoplasmic phosphoenolpyruvate carboxylase), NAD-cytMDH (cytosolic NAD-dependent malate dehydrogenase), l-Idn-DH (l-idonate dehydrogenase), mMDH (mitochondrial malate dehydrogenase), mNAD-ME (mitochondrial NAD-dependent malic enzyme), cytMDH (cytosolic malate dehydrogenase), cytNADP-ME (cytosolic MADP-dependent malic enzyme), PEPCK (phosphoenolpyruvate carboxykinase). (1) *VviSWEET10* (VIT_01s0146g00260); (2) *VviSWEET15* (VIT_17s0000g00830); (3) *VviSUC11* (VIT_18s0001g08210), *VviSUC12* (VIT_01s0026g01960); (4) *VviHT2* (VIT_18s0001g05570), *VviHT1* (VIT_00s0181g00010), *VviHT3* (VIT_11s0149g00050); (5) *VviHT6* (VIT_18s0122g00850); (6) *VviTMT2* (VIT03s0038g03940); (7) aquaporins (TIPs, NIPs, PIPs); (8) malate transporter.

Grape ripening is characterized by a double-sigmoid curve with two distinct growth phases separated by a phase of slow or no growth, termed the lag phase (Winkler and Williams, 1935). Table 1 summarizes critical processes involved in different stages of berry development. Following its synthesis in the source leaves, bulk (mass) flow in the phloem translocates sucrose towards the grape berry sinks. During early berry development, the berry unloads sucrose from the phloem symplastically via plasmodesmata, shifting at the onset of ripening to apoplastic unloading aided by transporters and invertases (Zhang *et al.*, 2006; Ren *et al.*, 2023) (Fig. 1). The metabolic and transcriptomic shift from berry development (phase I) at the end of the lag phase (phase II) marks the onset of berry ripening (phase III) (Coombe, 1992; Zhang *et al.*, 2006; Fortes *et al.*, 2015). The regulation of grape berry ripening is driven by an interplay of phytohormones during the distinct growth phases. After the berry set, cell division and cell expansion in berries are driven by auxin, cytokinin, and gibberellin, which reach high concentrations in this early phase (Conde *et al.*, 2007; Fortes *et al.*, 2015). As a non-climacteric fruit, the onset of ripening is assumed to be mainly controlled by a increase in abscisic acid (ABA) biosynthesis (Deluc *et al.*, 2007; Sun *et al.*, 2010; Pilati *et al.*, 2017). Apart from higher levels of ABA, brassinosteroids, and ethylene, greater sensitivity to ethylene signaling is documented in grape berries (Chervin *et al.*, 2004; Fortes *et al.*, 2015), suggesting a coordinated activity of these three phytohormones.

**Table 1.** Summary of the main physiological processes and metabolites involved in the double-sigmoid growth dynamics of grape berry development and ripening

	Growth phase I: berry development	Lag phase II: seed development	Growth phase III: berry ripening
Berry growth	Growth via cell division and cell expansion—berries green and hard [1]	Little or no berry growth	Start of berry softening at ripening start [1] Growth via cell expansion—activity of cell wall modification enzymes [1]
Vascular flow	Inflow of water, sugar, and nutrients via both the phloem and xylem [1]		Shift from symplastic to apoplastic phloem unloading [2] Inflow of water, sugar, and nutrients via phloem [3, 4] Excess water recycling through xylem [4]
Primary metabolites	Organic acids (tartaric and malic acids) accumulate in vacuoles [5]	Increase in activity of invertases, sucrose synthase, and sugar transporters [6]	Hexoses (up to 1.5 M) stored in vacuoles [7] Malic acid is metabolized, tartaric acid diluted through berry growth [8] Amino acids accumulate [8]
Secondary metabolites—polyphenols	Biosynthesis of hydroxycinnamates (phenolic acids) [5] Biosynthesis of flavan-3-ols [5, 15] Flavonol biosynthesis in berry skins as protection from UV light [9]	Biosynthesis of flavan-3-ols and flavonols [5, 9, 15]	Anthocyanin biosynthesis in berry skins of red grape varieties [9] Polymerization of flavan-3-ols [5, 15] Flavonol biosynthesis in berry skins [9]
Secondary metabolites—aroma compounds	Biosynthesis of monoterpenes and sesquiterpenes [10] Unsaturated fatty acids to form C_6_-aldehydes and alcohols [11] Biosynthesis of methoxypyrazines [11]	Biosynthesis of monoterpenes and sesquiterpenes [10] Biosynthesis of methoxypyrazines [11]	Accumulation of norisoprenoids from carotenoids, monoterpenes, sesquiterpenes, thiols (plus their conjugations) [12] Methoxypyrazines metabolized [11]
Phytohormones	Accumulation of auxins, cytokinins, gibberellins, and jasmonic acid [13]	Auxin delays onset of ripening, then declines; abscisic acid and ethylene signals accompany berry softening—coordination of start of ripening [14]	Gibberellins high in early phase of ripening—cell wall modification, role of cytokinins in sink strength [13]

[1] Coombe,1992; [2] Zhang *et al.*, 2006; [3] Keller *et al.*, 2006; [4] Keller *et al.*, 2015; [5] Conde *et al.*, 2007; [6] Walker *et al.*, 2021a; [7] Keller and Shrestha, 2014; [8] Dai *et al.*, 2013; [9] Allegro *et al.*, 2021; [10] Siebert *et al.*, 2018; [11] Robinson *et al.*, 2014; [12] Lin *et al.*, 2019; [13] Fortes *et al.*, 2015; [14] Fasoli *et al.*, 2018; [15] Gouot *et al.*, 2019.

It is well documented that berries on the same grape cluster ripen heterogenously (Coombe, 1992). The period for all berries within a cluster to change color at ripening onset can span 20–30 d (Hernández-Montes *et al.*, 2021). Recent studies determined distinct patterns in gene expression and phytohormone contents of berries at different ripening stages in the same cluster (Savoi *et al.*, 2021; Davies *et al.*, 2022), pointing towards a potential role for phytohormones, among other factors, in the asynchronicity of berry ripening. Morevoer, many processes at the onset of ripening occur in sequence rather than simultaneously. For example, berries soften mostly before sugar accumulation starts, and sugar accumulation, in turn, is a prerequisite for anthocyanin biosynthesis (Terrier *et al.*, 2005; Castellarin *et al.*, 2016; Hernández-Montes *et al.*, 2021). Molecular and biochemical evidence suggests that fruit softening occurs mainly through the relaxation and disassembly of mesocarp cell walls (Shi *et al.*, 2023). However, a decline in mesocarp cell turgor related to solute accumulation in the apoplast has been proposed as an alternative mechanism (Krasnow *et al.*, 2008). Berry softening is accompanied by cell separation in the mesocarp (Shi *et al.*, 2023). Nevertheless, the mesocarp cell membranes remain intact until late in the ripening phase (Krasnow *et al.*, 2008; Keller and Shrestha, 2014). When they finally begin to fail, cells in the locular region surrounding the seeds are the first to lose membrane integrity.

The beginning of sugar (primarily glucose and fructose) accumulation in the berry vacuoles is supported by a substantial increase in phloem influx, which is accompanied by a reversal of xylem flow as a means to discharge excess water derived from phloem influx (Keller *et al.*, 2015; Zhang and Keller, 2017).

The key steps of phloem unloading are summarized in Fig. 1. Briefly, when ripening initiates, the berries become symplastically isolated from the phloem, which facilitates apoplastic phloem unloading (Zhang *et al.*, 2006; Keller *et al.*, 2015), involving sugar transport across membranes mediated by proton-coupled sucrose transporters (SUTs; disaccharide transporters), hexose transporters (HTs; monosaccharide transporters), the passive facilitators SWEET (sucrose will eventually be exported transporters), and sugar metabolic enzymes such as acid invertases (AIs), neutral invertases (NIs), and sucrose synthases (SuSys) (Castellarin *et al.*, 2011; Zhang *et al.*, 2019; Walker *et al.*, 2021a). More precisely, sucrose, unloaded via VviSWEET10 (Zhang *et al.*, 2019; Savoi *et al.*, 2021) or sucrose transporters VviSUC12 and VviSUC11 (Afoufa-Bastien *et al.*, 2010; Lecourieux *et al.*, 2013), is hydrolyzed to glucose and fructose (cell wall invertase: VvicwINV), which are transported into the cytosol of parenchyma cells (VviSUC11/12, VviHT1–VviHT5, VviSWEET15, and VviSWEET10) (Hayes *et al.*, 2007; Zhang *et al.*, 2019; Ren *et al.*, 2020; Savoi *et al.*, 2021). Sucrose in pericarp cells provides major metabolic precursors by entering glycolysis through the activity of either neutral and acid invertases (cytINVs) (Nonis *et al.*, 2008) or SuSys to build UDP-glucose or ADP-glucose (Stein and Granot, 2019). Hexoses, and partly sucrose, are further transported into vacuoles through tonoplast monosaccharide transporters (VviTMT2 and VviHT6) or sucrose transporters (Afoufa-Bastien *et al.*, 2010; Ren *et al.*, 2023). As hexoses accumulate in the berry, previously accumulated malate temporarily becomes the substrate for respiration and gluconeogenesis (Sweetman *et al.*, 2009; Famiani *et al.*, 2016). Tartaric acid and malic acid accumulate in the vacuole during the first growth phase, and peak before the onset of berry ripening (Conde *et al.*, 2007). Tartaric acid, produced from the ascorbate biosynthetic pathways (Burbidge *et al.*, 2021), remains constant in content during ripening, while its concentration declines due to a dilution effect as the berry resumes expansive growth (Conde *et al.*, 2007; Hernández-Montes *et al.*, 2021). Malic acid biosynthesis has its starting point with glycolysis, leading to the decarboxylation of PEP (phosphoenolpyruvate) via PEPC (phosphoenolpyruvate carboxylase), resulting in oxaloacetate, which is processed to malate via NAD-cytMDH (cytosolic NAD-dependent malate dehydrogenase) (Ma *et al.*, 2018).

Akin to the primary metabolites, secondary metabolites accumulate in grape berries of both red and white cultivars at different developmental stages (Table 1). Unlike their white grape counterparts, the red grape cultivars accumulate anthocyanins in their skins, an essential trait for marketing table grapes and making red wine (He *et al.*, 2010). Anthocyanins and other phenolic compounds, such as flavanols and flavonols, are synthesized via the well-described phenylpropanoid and flavonoid pathway (He *et al.*, 2010; Qin *et al.*, 2022). Additionally, aroma compounds such as monoterpenes, sesquiterpenes, norisoprenoids, methoxypyrazines, lipoxygenase pathway products, and C6-aldehydes or alcohols and thiols accumulate in the berry mesocarp and/or skin cells before and/or during ripening (Lin *et al.*, 2019), imparting aroma and flavor to the wines following fermentation.

## Grapevine ripening disorders: distinct shriveling patterns of different shrivel types

This section provides information on the nomenclature of different shriveling disorders often mistaken for one another. Furthermore, it reviews the physiological background/mechanisms of the symptoms of BS, including the effects of genotype and environment.

### Symptoms of ripening disorders

**Fig. 2.**
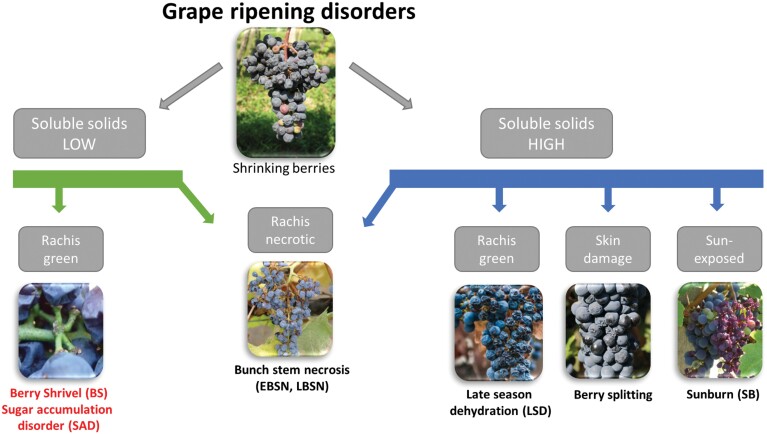
Main characteristics and differentiation of grapevine ripening disorders. Pictures by Griesser and Keller.

Grape berry ripening disorders differ in their timing of appearance and their underlying physiological and biochemical causes and consequences (Fig. 2). Yet, they all cause shriveling; however, the shriveling pattern is distinct in each disorder and on this basis can be classified as berry shrivel (BS), bunch stem necrosis (BSN), and late-season dehydration (LSD) (Krasnow *et al.*, 2010; Bondada and Keller, 2012b, 2017; Griesser *et al.*, 2012). LSD shows high sugar concentration in mature shriveled berries, whereas immature shriveled berries with low sugar concentration signify BS. BS- and LSD-affected fruits have a green and healthy cluster framework (peduncle, rachis, and pedicels); however, they become necrotic in BSN (Krasnow *et al.*, 2010; Bondada and Keller, 2012b). A similar phenomenon occurs in early bunch stem necrosis (EBSN; aka inflorescence necrosis), which develops in flower clusters before fruit set (Jackson and Coombe, 1988; Keller and Koblet, 1995). Sunburn injury has recently attracted more attention even in temperate climates due to rising temperatures, especially in berries directly exposed to high solar radiation following the common vineyard management practice of cluster-zone defoliation (Gambetta *et al.*, 2021). Berry splitting is a failure of the cuticle, and sometimes the epidermis, due to excess internal pressure, leading to dehydration in warm climates (Chang *et al.*, 2019; Chang and Keller, 2021). So far, no causal pathogens have been identified with any on the mentioned disorders.

Table 2 provides a synopsis of the grapevine ripening disorders: BS, BSN, LSD, and EBSN. BSN starts with small necrotic spots on the rachis surface, often near lenticels or stomata, which further spread into the rachis tissue, leading to a girdling effect blocking phloem transport (Hifny and Alleweldt, 1972; Brendel *et al.*, 1983; Hall *et al.*, 2010). In addition, tyloses may form in the xylem vessels in the necrotic region (Bondada and Keller, 2012a). Nevertheless, unlike phloem girdling, experimentally blocking xylem transport in the peduncle had no effect on berry growth and ripening (Hall *et al.*, 2010; Keller *et al.*, 2015), suggesting that the ensuing berry shriveling probably arises mostly as a result of berry transpiration (Zhang and Keller, 2015). Attempts have also been made to link BSN with carbon starvation, nutrient deficiency or imbalance, ammonium toxicity, and the amino acid metabolite putrescine (Hinfy and Alleweldt, 1972; Keller and Koblet, 1995; Holzapfel and Coombe, 1998; Capps and Wolf, 2000); however, these results remain inconclusive.

**Table 2.** Comparative summary of ripening disorders in grapevine: berry shrivel, late-season dehydration, sunburn, and bunch stem necrosis

	Berry shrivel	Late season dehydration	Sunburn	Bunch stem necrosis
Berry symptoms	Shrinking—comparable with deflated football [1, 2] Reduced berry weight [3, 4] Symptoms appear shortly after onset of ripening [10]	Shrinking symptoms appear before harvest [5]	Brown spots to complete desiccation [6]	Shrinking to complete desiccation during bloom (EBSN) or during ripening (LBSN) [3]
Total soluble solids (TSS)	Low (10–13 °Brix) due to arrested phloem influx [1, 3, 4, 7]	High (>24 °Brix) due to dehydration [8, 9]	Depends on symptom severity—often high due to dehydration [6, 9]	Variable but often high due to dehydration [3, 9]
Titratable acidity (TA) and organic acids	TA often higher due to dehydration [10] Tartaric and malic acid content per berry not changed [4]; oxalic and citric acid reduced [11]	No differences observed [8]	Depends on symptom severity—inconsistent results, reduced values observed [12]	Inconsistent results—reduction of malic and tartaric acid observed [1]; no change observed [11]
Amino acids	Most amino acids reduced; higher hydroxyproline [10], arginine, and alanine [3]	No differences observed [8]	NA	Inconsistent—changed in both directions [13]
Mineral nutrients	Low yeast-assimilable N, K^+^, and other nutrients [11, 19]; low K^+^ in rachis and pedicels [14]	NA	NA	Low Ca^2+^ and Mg^2+^—possible effect on cell wall formation in the rachis [15]; berries with high Ca^2+^ concentration [1]
Anthocyanins and other phenolics	Low anthocyanins, genes for biosynthesis delayed [4, 10]; elevated skin tannins [20]	No differences in anthocyanins observed [8]	Inconsistent anthocyanin results; elevated flavonols [9, 6]	Low anthocyanins when induced early [1]
Aroma compounds	NA	Elevated massoia lactone and γ-nonalactone, other volatiles decreased, e.g. 2-hexenol [8]	Reduced aroma compounds [6] Elevated antioxidant activity e.g. carotenoids, glutathione [6]	Altered aroma profile, e.g. elevated γ-nonalactone and β-damascenone (shrivel type unclear) [13]
Rachis and pedicels	Green, no symptoms [2]	Green, no symptoms [3]	Depends on berry symptom severity—from no symptoms to complete desiccation [12]	Necrotic sections; girdling effect and xylem blockage by tylosis, complete desiccation [1]
Causes	Unknown	Berry transpiration and xylem backflow [16, 17]	High UV radiation, high temperature [12]	Inconsistent results on nutrient imbalance, ammonium toxicity, or putrescine accumulation [5, 18]

[1] Bondada and Keller, 2012b; [2] Knoll *et al.*, 2010; [3] Krasnow *et al.*, 2010; [4] Savoi *et al.*, 2019; [5] Capps and Wolf, 2000; [6] Gambetta *et al.*, 2021; [7] Griesser *et al.*, 2018; [8] Chou *et al.*, 2018; [9] Bondada and Keller, 2017; [10] Griesser *et al.*, 2012; [11] Keller *et al.*, 2016; [12] Rustioni *et al.*, 2014; [13] Šuklje *et al.*, 2016; [14] Griesser *et al.*, 2017; [15] Christensen and Boggero, 1985; [16] Greer and Rogiers, 2009; [17] Tilbrook and Tyerman, 2008; [18] Holzapfel and Coombe, 1998; [19] Zufferey *et al.*, 2015; [20] Krasnow *et al.*, 2009. NA: not analyzed.

LSD of grape clusters occurs before harvest. Initial results indicate that the LSD shriveling results from dehydration via berry transpiration (Greer and Rogiers, 2009) and water backflow through the xylem, which may or may not be associated with a loss in mesocarp cell viability (Keller *et al.*, 2006; Tilbrook and Tyerman, 2008).

Sunburn symptoms range from brown or necrotic spots on berry skins to complete desiccation, depending on the intensity and duration of heat stress (Rustioni *et al.*, 2014). Berry temperatures >45–50 °C can induce brown and necrotic spots on berry skins due to oxidative stress, causing phenolic oxidation associated with cell decompartmentalization and possibly resulting in berry cracking and desiccation (Rustioni *et al.*, 2014, 2023; Müller *et al.*, 2023).

Berries affected by BS, the focus of this review, stop accumulating sugar, resulting in low total soluble solids (Keller *et al.*, 2016; Griesser *et al.*, 2018) and total anthocyanin content (Savoi *et al.*, 2019). Unlike in healthy berries that are often harvested at >20 °Brix, a plateau of soluble solids of 12–15 °Brix (corresponding to ~650–850 mM hexoses) is observed in BS berries of different grape cultivars (Bondada and Keller, 2012b, 2017; Griesser *et al.*, 2012; Savoi *et al.*, 2019; Hoff *et al.*, 2021). Initial research suggested that the arrest of sugar accumulation might be a consequence of cell death in the phloem of the cluster rachis, akin to a girdling effect (Hall *et al.*, 2010; Zufferey *et al.*, 2015). Recent work, however, showed that cell death starts in the berries, most commonly around the central vascular bundles proximal to the seeds, and may or may not progress to the pedicel and rachis (Hoff *et al.*, 2021). In either case, the cause of cell death remains unknown, and no other possible causes of BS induction have been observed. Once initiated, the impaired sugar import into the berries triggers downstream effects on primary and secondary metabolism before the visible symptoms of BS appear (Griesser *et al.*, 2018; Savoi *et al.*, 2019). Additionally, BS berries remain low in K^+^ (which, like sucrose, is imported via the phloem) and pH, and some reports showed a higher total acidity in BS berry juice (Bondada, 2014), possibly due to a concentration effect of less turgescent berries (Griesser *et al.*, 2012). Nevertheless, malate degradation in BS berries proceeds at a similar rate to that in healthy berries (Bondada and Keller, 2012a, b; Keller *et al.*, 2016).

### Berry shrivel phenotype: genotype and management effects

**Fig. 3.**
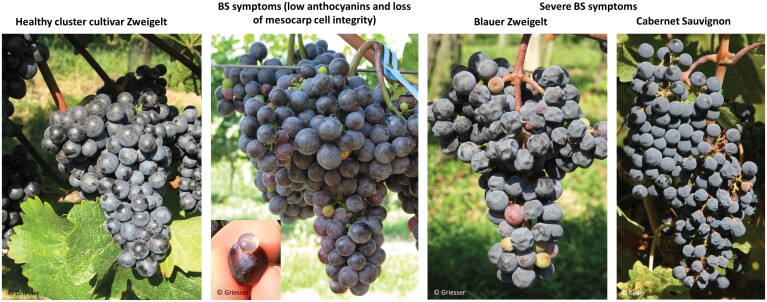
Berry shrivel (BS) symptom severity in *Vitis vinifera* cultivars Blauer Zweigelt and Cabernet Sauvignon in comparison with healthy clusters at harvest. BS symptoms evolve through berry ripening from berries with low anthocyanins to shriveled berries. Entire clusters are affected by BS, and detached berries leak juice from the mesocarp cells. Note the presence of a healthy cluster and a BS cluster on the same Cabernet Sauvignon shoot on the right. All pictures by Griesser and Keller.

BS berries gradually become flaccid and soft, indicating a collapse of the mesocarp, leaving the berries to appear like a deflated football (Bondada and Keller, 2017; Hoff *et al.*, 2021) (Fig. 3). The entire grape cluster, rather than individual berries, typically shows BS symptoms; however, earlier or more severe BS symptoms near the cluster tip sometimes occur relative to other cluster sections (Bondada, 2014; Hoff *et al.*, 2021). BS incidence in vineyards varies yearly, ranging from zero to 60%, with an afflicted vine showing both BS and healthy clusters (Krasnow *et al.*, 2009; Knoll *et al.*, 2010; Griesser *et al.*, 2012). Empirical evidence indicates that there is a genotype effect of BS incidence. Grapevine varieties that succumb to BS include Cabernet Sauvignon, Blauer Zweigelt, Pinot noir, Pinot blanc, Durif, Sémillon, Sauvignon blanc, Grüner Veltliner, Nebbiolo, Chasselas, Humagne rouge, Gewürztraminer, Melon, Merlot, and Cornalin, while other cultivars growing at the same vineyard sites remain unaffected. Quantitative data of BS occurrence are scare, but some studies tested possible environmental or vineyard management effects on BS. Different soil fertilization showed no effect on BS abundance in southern Germany with Blauer Zweigelt (22–29% BS) and Pinot blanc (5–6% BS) (Bachteler *et al.*, 2015b). In South Tirol, Italy, a severe reduction in canopy height (60 cm instead of 90 cm through repeated shoot topping) of Gewürztraminer, Pinot blanc, and Pinot gris led to a BS incidence of 20–40% compared with 5% in the untreated control (Raifer *et al.*, 2015). In western Switzerland, irrigation increased BS incidence compared with non-irrigated Humagne rouge (Zufferey *et al.*, 2015). The same study showed that strong temperature fluctuations near the onset of fruit ripening appeared to exacerbate the appearance of BS symptoms.

## Major metabolic shifts in sugar accumulation of berries afflicted with BS disorder

This section provides in-depth information on biochemical and morphological consequences of BS in berries, pedicels, and rachis. The focus will be on sugar transport and metabolism, cell death and cell wall modification, and phytohormone profiles.

### Primary and secondary metabolism are strongly affected

Previous studies identified major changes in primary and secondary metabolism prior to the appearance of visible symptoms of BS (Bondada and Keller, 2012a; Keller *et al.*, 2016; Griesser *et al.*, 2018, 2020). In addition, a transcriptional analysis found (i) no differential gene expression in BS berries before the onset of ripening; (ii) a high number of modulated genes from different metabolic pathways in BS berries at the onset of ripening; (iii) a high number of ‘switch’ genes showing reduced expression in BS berries at ripening onset; and (iv) enhanced activity of different metabolic pathways in BS berries with visible symptoms (Savoi *et al.*, 2019).

**Fig. 4.**
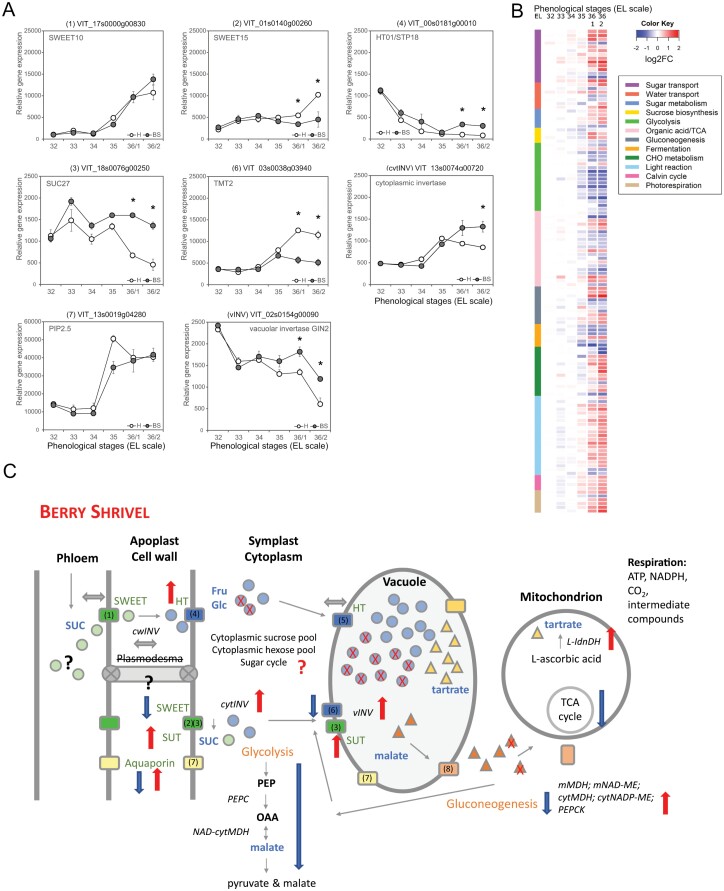
Illustrated summary of the processes affected in berry shrivel (BS) grapes with a focus on sugar transport and primary metabolism, and selected results of an RNA-seq study of related pathways. (A) Relative expression of genes involved in sugar transport and sugar metabolism differing between BS and healthy berries. (B) Heatmap of genes involved in sugar metabolism during berry development and ripening (data obtained from Savoi *et al.*, 2019). (C) Altered processes in BS berries with focus on sugar transport and primary metabolism. Enhanced processes in red, decreased processes in blue. Phenological (EL) stages: 32 (30 DAA days after anthesis), 33 (44 DAA), 34 (51 DAA), 35 (58 DAA), 36/1 (65 DAA), 36/2 (72 DAA). SUC (sucrose), Fru (fructose), Glc (glucose), PEP (phosphoenolpyruvate), OAA (oxalacetate), vINV (vacuolar invertase), cytINV (cytosolic invertase), cwINV (cell wall invertase), PEPC (cytoplasmic phosphoenolpyruvate carboxylase), NAD-cytMDH (cytosolic NAD-dependent malate dehydrogenase), l-Idn-DH (l-idonate dehydrogenase), mMDH (mitochondrial malate dehydrogenase), mNAD-ME (mitochondrial NAD-dependent malic enzyme), cytMDH (cytosolic malate dehydrogenase), cytNADP-ME (cytosolic MADP-dependent malic enzyme), PEPCK (phosphoenolpyruvate carboxykinase). (1) *VviSWEET10* (VIT_01s0146g00260), (2) *VviSWEET15* (VIT_17s0000g00830); (3) *VviSUC11* (VIT_18s0001g08210), *VviSUC12* (VIT_01s0026g01960), (4) *VviHT2* (VIT_18s0001g05570); *VviHT1* (VIT_00s0181g00010); *VviHT3* (VIT_11s0149g00050), (5) *VviHT6* (VIT_18s0122g00850), (6) *VviTMT2* (VIT03s0038g03940), (7) aquaporins (TIPs, NIPs, PIPs), (8) malate transporter.

In Fig. 4A–C, we summarize the available information on sugar transport and sugar metabolism in BS-affected grape berries during and after the onset of ripening. At the onset of normal grape ripening, the plasmodesmata are thought to be closed (Zhang *et al.*, 2006) and, therefore, sugars must enter the parenchyma cells via the apoplast (Fig. 1B). *VviSWEET10* and *VviHT6* of BS berries, with a proposed function in phloem unloading of sucrose into the apoplast and further transport to the vacuole of adjacent parenchyma cells (Zhang *et al.*, 2019; Savoi *et al.*, 2021), were not differentially expressed compared with healthy berries (Savoi *et al.*, 2019). Similarly, the cell wall invertase gene (*VvicwINV*) showed the same expression pattern in healthy and BS-affected berries. In contrast, the expression of the hexose transporter genes *VviHT1*, *VviHT4*, and *VviHT5*, and the sucrose transporter gene *VviSUC27* was induced in BS berries, which might have stimulated the relatively higher expression of two cytoplasm-neutral invertases. Additionally, the expression of the tonoplast monosaccharide transporter gene *VviTMT2* is highly reduced in BS berries, while the expression of the vacuolar invertase gene *VviGIN2* is enhanced (Savoi *et al.*, 2019). Together, these results could point towards a shift in the relative ratio of hexoses to sucrose in the apoplast and cytoplasm of mesocarp cells in BS berries, which could occur as a result of either changed transporter activities or imperfect symplast isolation via blocking of plasmodesmata. Alternatively, these changes might be a consequence of mesocarp membrane failure, a timely succession of events which needs elucidation. Despite altered expression of sugar transporter genes, no differences in the relative proportions of glucose, fructose, and sucrose were found in juice from BS berries compared with healthy berries (Keller *et al.*, 2016). Loss of membrane integrity in the cells surrounding the basal vascular bundles (or in the vascular bundles themselves) compromises the cells’ ability to osmoregulate and greatly reduces the berry’s sink activity and strength. The observed general shutdown of glycolysis and the tricarboxylic acid (TCA) cycle (Fig. 4B) may be a consequence of the lack of glucose precursors in BS berries (Griesser *et al.*, 2018; Savoi *et al.*, 2019). Strikingly, however, the altered expression of genes in BS berries at the onset of ripening cannot explain the cessation of sugar accumulation shortly thereafter. Post-transcriptional processes could modify expression levels, as shown for the sucrose transporter SUC2 in leaves via ubiquitination and phosphorylation (Xu *et al.*, 2020).

Apart from low sugar content, the pH of juice from BS berries is more acidic than that of normally ripening berries (Krasnow *et al.*, 2009; Bondada and Keller, 2012b; Griesser *et al.*, 2012). This difference is likely to be a result of higher concentrations of titratable acidity due to berry shrinkage (Zufferey *et al.*, 2015) and decreased import of K^+^ via the phloem (Keller *et al.*, 2016). Information on the organic acid profile in BS berries is limited, as most studies focused on tartrate and malate, which showed similar values for tartrate and slightly lower or unchanged values for malate (Krasnow *et al.*, 2009; Bondada and Keller, 2012a). One study determined lower amounts of citrate and, in particular, oxalate, in BS berries (Keller *et al.*, 2016). As tartaric and malic acid accumulate in growth phase I, the observed organic acid and transcriptional profile in BS berries supports the idea that BS does not affect early berry development, at least not organic acid biosynthesis. The expression of genes related to the TCA cycle and associated pathways (e.g. pyruvate kinase, an enolase/phosphopyruvate hydratase, a glyceraldehyde-3P dehydrogenase, or a cytosolic NADP-dependent malic enzyme) was suppressed in BS berries at the onset of ripening (Fig. 4B). Consequently, reduced availability of precursors may have affected the biosynthesis of flavonoids, as the expression of most structural genes was decreased (Griesser *et al.*, 2018; Savoi *et al.*, 2019). Nonetheless, the branch leading to flavanol production was up-regulated, resulting in lower anthocyanin and higher skin tannin contents in BS berries at harvest (Krasnow *et al.*, 2009; Savoi *et al.*, 2019). Whether this observed pattern is the result of a direct competition between anthocyanin and flavanol biosynthesis, as shown for strawberries (Fischer *et al.*, 2014), or whether it is based on other mechanisms remains to be determined. However, the delayed and reduced anthocyanin biosynthesis could result from the low sugar contents, as sugar sensing and signaling stimulate anthocyanin biosynthesis (Lecourieux *et al.*, 2013). A concentration of ~500 mM total sugars is needed in grape berries to start anthocyanin biosynthesis (Dai *et al.*, 2014; Keller and Shrestha, 2014).

In summary (Fig. 4C), analytical and transcriptional results show that although sugar accumulation in BS berries starts at the onset of ripening, the process quickly slows and stops during early berry ripening. The arrest in sugar import has consequences for anthocyanin biosynthesis and other primary and secondary metabolic pathways. The sugar cycle and organic acid valve in cells are tightly coordinated throughout grape berry development and ripening, and it needs to be determined if the cessation of sugar accumulation is one of the first symptoms or the cause of BS development.

### Cell wall modification, cell death, and callose deposition may impair assimilate transport and reactive oxygen species scavenging

Much of our current knowledge of BS originates from microscopic studies of the vascular system in the rachis and pedicels (Zufferey *et al.*, 2015; Crespo-Martínez *et al.*, 2019), transcriptome analysis in the rachis and berries (Savoi *et al.*, 2022), and the investigation of cell death (i.e. membrane failure) in the rachis, pedicels, and berries (Hall *et al.*, 2010; Hoff *et al.*, 2021). An in-depth microscopic study of the pedicel–receptacle–brush junction in BS berries is pending. The brush, an opaque flesh consisting of vascular tissues in healthy berries, remains attached to the pedicel when the berry and pedicel are pulled apart. In contrast, the brush lacks flesh when BS berries are removed (Bondada and Keller, 2012a; Hoff *et al.*, 2021), which is consistent with the notion that cell death in the BS syndrome starts in the brush area. At advanced stages of grape ripening, a higher percentage of cell death is observed near the seeds in the inner mesocarp, even in healthy berries (Krasnow *et al.*, 2008; Tilbrook and Tyerman, 2008). Development-related programmed cell death (PCD) has substantial regulatory functions in cell differentiation, biological development, and senescence of organs, with reactive oxygen species (ROS) as common inducers and molecular signals, as has been reviewed (Ye *et al.*, 2021). A change in plant vacuolar membrane permeability is a marker of PCD initiation as various hydrolases are released (Chichkova *et al.*, 2004). The mechanisms of the cell death process at late berry ripening stages are poorly understood. One factor that may contribute to the enhanced cell death is low oxygen levels in berry mesocarp, especially near the seeds (Xiao *et al.*, 2018).

**Fig. 5.**
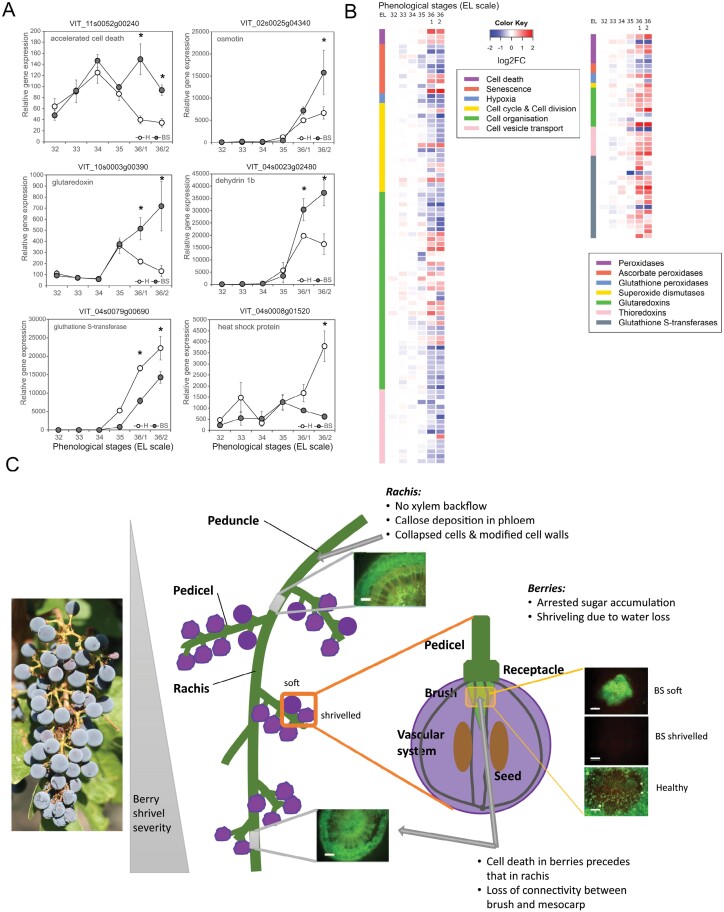
Illustrated summary of the current knowledge of the induction of berry shrivel (BS) in grapes with a focus on the vascular system, and the cell wall and cell death processes. (A) Relative expression of genes involved in cell death and ROS metabolism differing between BS and healthy berries. (B) Heatmap of genes involved in cell death, cell organization, and ROS scavenging (data obtained from Savoi *et al.*, 2019). (C) Cell death in rachis, pedicels, and berries. The presented results were obtained from Krasnow *et al.* (2009), Hall *et al.* (2010), Bondada and Keller (2012a), Bondada (2014), Crespo-Martínez *et al.* (2019), and Hoff *et al.* (2021). Phenological (EL) stages: 32 (30 DAA days after anthesis), 33 (44 DAA), 34 (51 DAA), 35 (58 DAA), 36/1 (65 DAA), 36/2 (72 DAA).

Many signals integrate into a death or survival response in plant cells, including Ca^2+^ and ROS, external and intracellular receptors, and kinases, and increasing evidence points towards phytohormones as regulating factors (Ye *et al.*, 2021). Ethylene is assumed to trigger and promote PCD, and ABA is a well-known positive regulator of leaf senescence. At the same time, cytokinins prolong the greenness period in leaves, thereby delaying senescence (Ye *et al.*, 2021). Indeed, the expression of three genes associated with cell death and several markers for oxidative stress (osmotin, glutaredoxin, and thioredoxin) were increased in BS berries (Fig. 5A). At the same time, some peroxidases and one glutathione *S*-transferase (GST) were suppressed (Fig. 5A, B). The expression of GSTs is often enhanced under biotic and abiotic stress conditions in parallel with the production and accumulation of ROS, which led to the idea that GSTs have a protective role against oxidative stress apart from their known role in the detoxification of exogeneous xenobiotics or intracellular oxidized molecules, and in anthocyanin transport to vacuoles (Ugalde *et al.*, 2021). Consequently, ROS accumulation could induce the observed cell death and enhanced expression of genes associated with redox processes in BS symptomatic berries (Fig. 5B). Additionally, the expression of 20 heat shock proteins (HSPs) is suppressed in BS berries, while only three HSPs are enhanced (Fig. 5A). HSPs are stress-responsive molecules with a primary function in proper protein folding, unfolding, and transport, thereby supporting membrane stability and ROS-scavenging enzyme activity (Ul Haq *et al.*, 2019). Their failed induction might trigger the observed enhanced cell death.

Apart from cell death, callose plugging of sieve tubes has been observed in the rachis of both healthy and BS-afflicted clusters, but the extent of the plugging is much greater in grape clusters showing BS symptoms (Bondada and Keller, 2012a). However, manipulating the vascular system by selective xylem or phloem removal in the peduncle at the onset of ripening failed to induce BS (Hall *et al.*, 2010; Hoff *et al.*, 2021). Future work should determine whether callose deposition occurs as part of a wound response that accompanies cell death in the phloem. In addition to callose deposition, collapsed phloem cells have been observed in the proximity of reduced cambium cell layers (Zufferey *et al.*, 2015; Crespo-Martínez *et al.*, 2019). The reduced expression of cell wall biosynthesis genes and genes involved in different secondary metabolic processes in BS rachis shows parallels to a sugar starvation response in Arabidopsis (Arias *et al.*, 2014; Savoi *et al.*, 2022).

In summary (Fig. 5), berries with BS symptoms show premature cell death in the brush region, which may be induced by oxidative stress or a failed regulatory mechanism to scavenge ROS, and may or may not spread to the pedicel and the rachis. As the brush area includes and connects the vascular tissues of berries and pedicels, its failure may interrupt both the assimilate transport in the phloem towards the berries and water backflow in the xylem from the berries. However, why and how these changes occur virtually simultaneously in all or most berries of a grape cluster, despite their otherwise asynchronous development, remains to be determined.

### Regulation of grape ripening as a possible factor in BS induction

Phytohormones, specifically ABA, control ripening in non-climacteric fruit species such as grapes; auxin, brassinosteroids, and ethylene also play a significant role (Davies and Böttcher, 2009). The onset of ripening in grape berries is characterized by a transcriptional reprogramming, including a small group of genes (called ‘switch’ genes) changing their expression level from low in green immature berries to high in ripening berries (Palumbo *et al.*, 2014; Fasoli *et al.*, 2018). In BS berries, a suppression of the majority of switch genes was observed at the onset of ripening (Savoi *et al.*, 2019). The induction of some of these genes was delayed (e.g. *VviGST4*, *VviUFGT*, *VviMYBA3*, or an alcohol dehydrogenase gene), while some genes failed to be induced in BS berries (e.g. a glyceraldehyde-3-P-dehydrogenase gene, an enolase gene, or a different alcohol dehydrogenase gene). Further studies must elucidate if the observed pattern is a mistiming of ripening onset, or the response to a specific stress situation induced by an as yet unknown factor. Despite the general transcriptional suppression at ripening onset, expression of some switch genes was enhanced in BS berries (e.g. *VviEXPB04*, *VviXTH32*, and *VviNAC60*) (Savoi *et al.*, 2019). Switch genes are assumed to be regulated by ABA (Pilati *et al.*, 2017), although no correlation between gene suppression and the active form of ABA in BS berries was observed (Griesser *et al.*, 2020). Nevertheless, an up-regulation of genes involved in ABA biosynthesis (*VviNCED2* and *VviNCED4*), ABA signaling (e.g. *VviPYL4* and *VviPYR1*), and ABA catabolism (e.g. *VviBGLU44*) was determined in symptomatic BS berries (Savoi *et al.*, 2019). Two weeks before the onset of ripening, an almost 3-fold increase of 1-aminocyclopropane-1-carboxylate (ACC), the ethylene precursor, was determined in BS berries (Griesser *et al.*, 2020). With current knowledge, the role of high levels of ACC in developing BS berries remains unclear; however, none of the studies applying phytohormones to manipulate grape berry ripening reported symptoms similar to BS (Böttcher *et al.*, 2013, 2022; Coelho *et al.*, 2019). The complex phytohormone crosstalk in ripening regulation and the regulation of cell death and senescence via phytohormones could be a reason for non-conclusive results.

In summary, a cascade of events could accumulate triggers for BS induction at ripening onset. Many modulated genes, and significantly suppressed switch genes in pre-symptomatic BS berries, suggest a disturbed ripening onset, while expression profiles in symptomatic BS berries change in both directions.

## Research achievements, hypotheses, and open questions

BS is a ripening disorder, and we do not know what causes it. However, substantial knowledge has accumulated recently, allowing us to target more specific questions to shed light on the induction process and symptom development. The highlights of the leading research achievements are as follows: (i) BS initiates at the onset or shortly after the onset of ripening in affected berries and quickly spreads to entire fruit clusters (Keller *et al.*, 2016; Griesser *et al.*, 2018); (ii) initial symptoms include loss of cell membrane integrity in the brush vascular area between the seeds and the receptacle, and an arrest of sugar import (Krasnow *et al.*, 2009; Hoff *et al.*, 2021); (iii) BS is associated with major shifts in primary and secondary metabolism at the transcriptional level, resulting in distinct metabolic profiles (Savoi *et al.*, 2019; Griesser *et al.*, 2020); (iv) in BS berries, switch genes are suppressed at the onset of ripening (Savoi *et al.*, 2019); (v) cell death in the mesocarp may spread to the phloem of the pedicel and the rachis (Hall *et al.*, 2010; Hoff *et al.*, 2021), and culminates in berry shrinkage from evaporative water loss; (vi) genes involved in cell wall modification and hydrolytic enzymes are differentially expressed in both berries and rachis (Savoi *et al.*, 2022); (vii) attempts to induce BS by selective xylem or phloem removal in the peduncle have been unsuccessful (Hall *et al.*, 2010; Hoff *et al.*, 2021); (viii) anatomical studies confirmed collapsed secondary phloem cells and callose deposition at sieve plates in the rachis (Bondada and Keller, 2012a, 2017; Crespo-Martínez *et al.*, 2019); and (ix) attempts to identify pathogens involved in BS induction have been unsuccessful (Krasnow *et al.*, 2009; Keller *et al.*, 2016).

Putting the observed biochemical, transcriptional, anatomical, and morphological changes involved in BS induction and symptom development into a spatial and temporal context in the rachis, pedicels, and berries on individual grape clusters or vines poses a challenge to sampling strategies. Samples from pre-symptomatic clusters need to be collected without disturbing the ripening process or the process of BS induction. Moreover, the sampling process must consider the added complexity imposed by the asynchronous onset of berry ripening. Based on the information gathered in this review, we propose three hypotheses for future research.

### Hypothesis 1

Hypothesis 1 encompasses the most prominent symptom of BS: the arrest of sugar accumulation early during grape ripening. The ripening program starts on time, as shown by the expression of sugar transporters and the initial increase in sugar content and decline in acidity. However, some events prevent its proper coordination, which may have consequences for the osmoregulation of mesocarp cells. The starting point could also be a failed symplastic isolation of the grape berry from the phloem, which would lead to inhibition of phloem unloading as the sugar concentration in the mesocarp cells begins to increase. Consequently, the berries on an affected cluster would fail to increase sink strength, but they would sustain a baseline support via the symplast until cellular membranes begin to fail. Therefore, hypothesis 1 states that BS is initiated by a failure in phloem unloading of sugar or its transport within the berry cells, resulting in osmotic stress in the cytosol. These processes would then compromise membrane integrity and anthocyanin biosynthesis, and would culminate in berry shrinkage via water loss through transpiration and xylem backflow.

### Hypothesis 2

Hypothesis 2 states that premature cell death in the brush area of berries initiates a runaway process of PCD towards other berry areas and the pedicels. Stress-induced ROS may trigger the response, damaging cell membranes and impending metabolism. Affected cells would fail to fulfill their sink function, which may result in a feedback loop hampering phloem unloading processes or vascular flow in general, ultimately resulting in arrested sugar accumulation. At some point, ROS production and ROS scavenging may be back in balance, as berries do not desiccate completely and induce ROS-scavenging transcriptional processes. In this scenario, the arrest of sugar accumulation would result from the loss of membrane integrity in the parenchyma cells surrounding the basal vascular bundles, or in the vascular bundles themselves, and perhaps of the connectivity to or within the vascular system.

### Hypothesis 3

Hypothesis 3 states that the berry ripening process in general is disturbed. This hypothesis may not be independent from hypothesis 1 and/or hypothesis 2. About three times more genes are suppressed than induced in pre-symptomatic BS berries at the onset of ripening, among them a high number of switch genes. A specific metabolic process within switch genes could not be identified. Currently, the seemingly random distribution across grape clusters of asynchronously ripening berries has not been considered in these studies, but BS symptoms often appear simultaneously (or nearly so) across a cluster. However, if ripening regulation is mistimed, there would be consequences for sink strength and for assimilate partitioning, resulting in reduced sugar accumulation and loss of cell vitality, both processes influenced by phytohormones.

Despite considerable progress in our understanding of BS, there are some puzzling facts about this ripening disorder, posed as questions below.

### Why does BS usually affect whole clusters rather than individual berries?

Initial metabolic changes in BS berries are observed at or shortly after the onset of berry ripening. Berries of the same cluster ripen quite heterogeneously (Coombe, 1992; Bigard *et al.*, 2019; Hernández-Montes *et al.*, 2021), and individual berries, rather than whole clusters, trigger enhanced phloem import as each berry begins to ripen over a period that can span >2 weeks (Keller *et al.*, 2015; Zhang and Keller, 2017). Many factors, including the timing of flowering and fruit set, berry growth, seed number, size, and content, contribute to asynchronous ripening (Coombe, 1992; Gouthu and Deluc, 2015; Keller *et al.*, 2022). Consequently, one factor for BS induction is still missing, as berries start ripening individually, but BS symptoms often appear across whole clusters simultaneously. There could be a signal for communication among berries of the same cluster that synchronizes the appearance of BS, but the nature of this signaling is yet to be deciphered. It is possible that an external signal from the vine or the environment might induce BS.

### Why are only some clusters on a vine or even on the same shoot affected?

The appearance of BS is random on vines, within vineyards, and between years. There is no obvious explanation for why some grape clusters on a shoot or a vine are affected while others remain healthy, or why some vines have clusters with BS symptoms while others produce wholly healthy grapes. The premise that BS is a consequence of potassium deficiency could not be experimentally validated (Bachteler *et al.*, 2015a, b; Griesser *et al.*, 2017), compelling us to explore soil, climate, or micro-climate factors as potential causes, as growers observe BS under varied growing conditions. The elucidation of potential environmental triggers could help to understand the patchiness of BS incidence, possibly by determining inflorescence development and fruit set or ripening onset. Since BS symptoms appear only during fruit ripening, after seeds have developed and matured, the putative environmental trigger does not influence stages I and II of berry growth. However, some changes in inflorescence development could enhance the BS probability later in the season. Since not all clusters are affected, a cluster-specific signal might lead to the induction of BS and the next question.

### Why are some grape cultivars more affected than others?

Not all grapevine cultivars are similarly affected by ripening disorders in general and BS specifically. Genotype–environment interactions could be one piece of the puzzle yet to be studied. Genetic pedigrees do not show close relationships between susceptible cultivars, for example Cabernet Sauvignon and Blauer Zweigelt. However, comparative studies investigating BS with several cultivars are scarce, and BS monitoring has not been included in routine assessments in cultivar collections. The seasonal timing of ripening initiation is probably not a factor in BS induction, as both early (Pinot noir) and late (Cabernet Sauvignon) ripening cultivars are affected. Similarly, anthocyanin biosynthesis is not involved in triggering BS, as both red and white cultivars are affected.

### Why do BS-affected berries remain metabolically active and not desiccate completely?

Although we observe reduced sugar accumulation and localized cell death in BS berries, the berries continue to metabolize malate, and many genes are differentially regulated. Moreover, although they shrink following the arrest of sugar accumulation, they do not desiccate completely and are not abscised from the cluster. This behavior is distinct from BSN, wherein large parts of the peduncle and/or rachis become necrotic, and the berries downstream of the necrotic sections dry up and shrivel. The transcriptomic activity provides a real-time snapshot of the cellular activity that may present a flux rather than homeostasis. Thus, the information extracted requires interpretation ‘with care’.

### Is there a linkage between the BSN and BS disorders?

The ripening disorders BS and BSN are usually distinguished from each other by their symptoms on the rachis and pedicels, which remain green in BS clusters but become necrotic in BSN clusters. Nevertheless, transitional forms have been observed, with small necrotic lesions visible under the microscope in rachis axils or pedicels of BS clusters. It is possible that the seemingly distinct disorders might represent differences in the degree of severity. In this scenario, BS would be a ‘mild’ form of BSN. However, this idea is difficult to reconcile with the observation that cell death may start within berries in BS and near rachis lenticels or stomata in BSN. It remains to be investigated whether BS and BSN are related via external or internal triggers.

## Outlook and future perspectives

Fruit ripening is a critical developmental phase of plant life, primarily for dispersing the seeds. However, we grow fruits to supplement our diet since fresh fruits and their processed products are enriched with health-promoting metabolites. These metabolites are the culmination of a well-orchestrated ripening program, the most studied phenomenon of grapevine life. Although fruit ripening has been studied extensively, especially in model species such as tomato and grapevine, many ripening aspects still need to be clarified because they result from complex mechanisms regulated by myriad abiotic and biotic factors underlying fruit ripening. Like other fruit species, grapevines continue to suffer from physiological ripening disorders, including BS, for which we have no remedy. BS afflicts grape berries every growing season to a varying degree, and in some years causes significant losses. This review provides a current understanding of this anomaly, including key challenges and critical knowledge gaps. New and innovative ideas are needed to answer the open questions and identify the causal processes that induce BS. Future research should prioritize the following areas. (i) Consolidating the available information on BS with different cultivars at the biochemical and transcriptional levels. Most studies to date focused on either Cabernet Sauvignon or Blauer Zweigelt. However, the two cultivars have never been compared side by side in the same study, which would help to identify cultivar-specific responses. The genetic background and parent–progeny associations of cultivars developing BS could add a different angle to the study of this phenomenon. Additionally, sampling protocols need to be standardized to ensure comparability of results. (ii) Jointly analyzing the assimilate transport towards grape berries and the metabolic processes within the berries. This analysis would include the heterogeneity of berry populations at the onset of ripening, the communication between berries and the signaling between berries and vines, and the consequences of vascular transport capacities for ripening processes. One focus should be on the pedicel–receptacle–brush–berry junction to directly trace phloem and xylem transport with labeled molecules. Quantifying phloem flow directly in living tissues would be interesting, but a method has yet to become available. The analyses of miRNA or siRNA signals in the vascular system might provide information on source–sink communication. (iii) Designing innovative experimental protocols aimed at inducing BS. Progress in elucidating potential underlying causes of this disorder is greatly hampered by the current inability to trigger BS in a laboratory setting or in the field. (iv) Pathogens have not been associated with BS, but that does not exclude the possibility that the disorder might be caused by an unknown pathogen. Also, studying endophytes may contribute to the current picture of the seasonality of BS abundance. (v) Developing strategies to reduce BS incidence in vineyards and clarifying the contribution of potential environmental triggers. Developing a database to track BS incidence in wine-growing regions would help to understand the severity of BS in viticulture. Linking the BS incidence on a spatial scale with remote sensing information, microclimatic measurements, vineyard management practices, and local soil characteristics may facilitate the identification of BS risk factors.

## Data Availability

All raw transcriptomics reads have been previously deposited (SRA (http://www.ncbi.nlm.nih.gov/sra) PRJNA436693 and SRP134067). Data presented in figures of this review are available at Zenodo https://doi.org/10.5281/zenodo.10003017; Griesser and Savoi (2023).

## References

[CIT0001] Afoufa-Bastien D , Medici A, Jeauffre J, Coutos-Thévenot P, Lemoine R, Atanassova R, Laloi M. 2010. The *Vitis vinifera* sugar transporter gene family: phylogenetic overview and macroarray expression profiling. BMC Plant Biology 10, 245. 21073695 10.1186/1471-2229-10-245 PMC3095327

[CIT0002] Allegro G , Pastore C, Valentini G, Filippetti I. 2021. The evolution of phenolic compounds in *Vitis vinifera* L red berries during ripening: analysis and role on wine sensory—a review. Agronomy 11, 999.

[CIT0003] Arias MC , Pelletier S, Hilliou F, Wattebled F, Renou J-P, D’Hulst C. 2014. From dusk till dawn: the *Arabidopsis thaliana* sugar starving responsive network. Frontiers in Plant Science 5, 482. 25295047 10.3389/fpls.2014.00482 PMC4170100

[CIT0004] Bachteler K , Riedel M, Merkt N, Schies W, Fröhlin J, Wünsche J. 2015a. Effects of foliar fertilization on incidence of berry shrivel and bunch stem necrosis in *Vitis vinifera* L cvs ‘Pinot Blanc’ and ‘Zweigelt’. Journal of Plant Nutrition 38, 839–853.

[CIT0005] Bachteler K , Riedel M, Merkt N, Ullrich B, Erhardt M, Wünsche JN. 2015b. Effect of soil fertilization on the incidence of berry shrivel and the quality of resulting wine. Vitis 52, 1–7.

[CIT0006] Bigard A , Romieu C, Sire Y, Veyret M, Ojéda H, Torregrosa L. 2019. The kinetics of grape ripening revisited through berry density sorting. OENO One 53, 709–724.

[CIT0007] Blancquaert EH , Oberholster A, Ricardo-da-Silva JM, Deloire AJ. 2019. Grape flavonoid evolution and composition under altered light and temperature conditions in Cabernet Sauvignon (*Vitis vinifera* L). Frontiers in Plant Science 10, 1062. 31798597 10.3389/fpls.2019.01062 PMC6874162

[CIT0008] Bondada B. 2014. Structural and compositional characterization of suppression of uniform ripening in grapevine: a paradoxical ripening disorder of grape berries with no known causative clues. Journal of the American Society for Horticultural Science 139, 567–581.

[CIT0009] Bondada B , Keller M. 2012a. Morphoanatomical symptomatology and osmotic behavior of grape berry shrivel. Journal of the American Society for Horticultural Science 137, 20–30.

[CIT0010] Bondada B , Keller M. 2012b. Not all shrivels are created equal—morpho-anatomical and compositional characteristics differ among different shrivel types that develop during ripening of grape (*Vitis vinifera* L) berries. American Journal of Plant Sciences 3, 879–898.

[CIT0011] Bondada B , Keller M. 2017. Structural and fruit compositional anomalies related to various shrivel types developing during ripening of grape berries. Acta Horticulturae 1157, 49–54.

[CIT0012] Böttcher C , Burbidge CA, Boss PK, Davies C. 2013. Interactions between ethylene and auxin are crucial to the control of grape (*Vitis vinifera* L) berry ripening. BMC Plant Biology 13, 222. 24364881 10.1186/1471-2229-13-222 PMC3878033

[CIT0013] Böttcher C , Johnson TE, Burbidge CA, Nicholson EL, Boss PK, Maffei SM, Bastian SEP, Davies C. 2022. Use of auxin to delay ripening: sensory and biochemical evaluation of Cabernet Sauvignon and Shiraz. Australian Journal of Grape and Wine Research 28, 208–217.

[CIT0014] Brendel G , Stellwaag-Kittler F, Theiler R. 1983. Die patho-physiologischen Kriterien der Stiellähme. Mitteilungen Klosterneuburg 33, 100–104.

[CIT0015] Burbidge CA , Ford CM, Melino VJ, et al. 2021. Biosynthesis and cellular functions of tartaric acid in grapevines. Frontiers in Plant Science 12, 643024. 33747023 10.3389/fpls.2021.643024 PMC7970118

[CIT0016] Capps ER , Wolf TK. 2000. Reduction of bunch stem necrosis of Cabernet Sauvignon by increased tissue nitrogen concentration. American Journal of Enology and Viticulture 51, 319–328.

[CIT0017] Castellarin SD , Gambetta GA, Wada H, Krasnow MN, Cramer GR, Peterlunger E, Shackel KA, Matthews MA. 2016. Characterization of major ripening events during softening in grape: turgor, sugar accumulation, abscisic acid metabolism, colour development, and their relationship with growth. Journal of Experimental Botany 67, 709–722. 26590311 10.1093/jxb/erv483 PMC4737070

[CIT0018] Castellarin SD , Gambetta GA, Wada H, Shackel KA, Matthews MA. 2011. Fruit ripening in *Vitis vinifera*: spatiotemporal relationships among turgor, sugar accumulation, and anthocyanin biosynthesis. Journal of Experimental Botany 62, 4345–4354. 21586429 10.1093/jxb/err150 PMC3153685

[CIT0019] Cataldo E , Salvi L, Paoli F, Fucile M, Mattii GB. 2021. Effect of agronomic techniques on aroma composition of white grapevines: a review. Agronomy 11, 2027.

[CIT0020] Chang BM , Keller M. 2021. Cuticle and skin cell walls have common and unique roles in grape berry splitting. Horticulture Research 8, 168. 34333518 10.1038/s41438-021-00602-2 PMC8325674

[CIT0021] Chang BM , Zhang Y, Keller M. 2019. Softening at the onset of grape ripening alters fruit rheological properties and decreases splitting resistance. Planta 250, 1293–1305. 31254101 10.1007/s00425-019-03226-y

[CIT0022] Chervin C , El-Kereamy A, Roustan J-P, Latché A, Lamon J, Bouzayen M. 2004. Ethylene seems required for the berry development and ripening in grape, a non-climacteric fruit. Plant Science 167, 1301–1305.

[CIT0023] Chichkova NV , Kim SH, Titova ES, Kalkum M, Morozov VS, Rubtsov YP, Kalinina NO, Taliansky ME, Vartapetian AB. 2004. A plant caspase-like protease activated during the hypersensitive response. The Plant Cell 16, 157–171. 14660804 10.1105/tpc.017889 PMC301402

[CIT0024] Chou HC , Šuklje K, Antalick G, Schmidtke LM, Blackman JW. 2018. Late-season Shiraz berry dehydration that alters composition and sensory traits of wine. Journal of Agricultural and Food Chemistry 66, 7750–7757. 29962206 10.1021/acs.jafc.8b01646

[CIT0025] Christensen LP , Boggero JD. 1985. A study of mineral nutrition relationships of waterberry in Thompson Seedless. American Journal of Enology and Viticulture 36, 57–64.

[CIT0026] Coelho J , Almeida-Trapp M, Pimentel D, Soares F, Reis P, Rego C, Mithöfer A, Fortes AM. 2019. The study of hormonal metabolism of Trincadeira and Syrah cultivars indicates new roles of salicylic acid, jasmonates, ABA and IAA during grape ripening and upon infection with *Botrytis cinerea*. Plant Science 283, 266–277. 31128697 10.1016/j.plantsci.2019.01.024

[CIT0027] Conde C , Silva P, Fontes N, Dias ACP, Tavares RM, Sousa MJ, Agasse A, Delrot S, Geros H. 2007. Biochemical changes throughout grape berry development and fruit and wine quality. Food 1, 1–22.

[CIT0028] Coombe BG. 1992. Research on development and ripening of the grape berry. American Journal of Enology and Viticulture 43, 101–110.

[CIT0029] Coombe BG , McCarthy MG. 2000. Dynamics of grape berry growth and physiology of ripening. Australian Journal of Grape and Wine Research 6, 131–135.

[CIT0030] Cramer GR , Cochetel N, Ghan R, Destrac-Irvine A, Delrot S. 2020. A sense of place: transcriptomics identifies environmental signatures in Cabernet Sauvignon berry skins in the late stages of ripening. BMC Plant Biology 20, 41. 31992236 10.1186/s12870-020-2251-7 PMC6986057

[CIT0031] Crespo-Martínez S , Sobczak M, Różańska E, Forneck A, Griesser M. 2019. The role of the secondary phloem during the development of the grapevine Berry Shrivel ripening disorder. Micron 116, 36–45. 30292168 10.1016/j.micron.2018.09.012

[CIT0032] Dai ZW , Léon C, Feil R, Lunn JE, Delrot S, Gomès E. 2013. Metabolic profiling reveals coordinated switches in primary carbohydrate metabolism in grape berry (*Vitis vinifera* L.), a non-climacteric fleshy fruit. Journal of Experimental Botany 64, 1345–1355. 23364938 10.1093/jxb/ers396 PMC3598422

[CIT0033] Dai ZW , Meddar M, Renaud C, Merlin I, Hilbert G, Delrot S, Gomès E. 2014. Long-term in vitro culture of grape berries and its application to assess the effects of sugar supply on anthocyanin accumulation. Journal of Experimental Botany 65, 4665–4677. 24477640 10.1093/jxb/ert489 PMC4115254

[CIT0034] Dai ZW , Vivin P, Barreuz F, Ollat N, Delrot S. 2010. Physiological and modelling approaches to understand water and carbon fluxes during grape berry growth and quality development: a review. Australian Journal of Grape and Wine Research 16, 70–85.

[CIT0035] Davies C , Böttcher C. 2009. Hormonal control of grape berry ripening. In: Roubelakis-Angelakis KA, ed. Grapevine molecular physiology & biotechnology. Dordrecht: Springer Netherlands, 229–261.

[CIT0036] Davies C , Robinson SP. 1996. Sugar accumulation in grape berries. Cloning of two putative vacuolar invertase cDNAs and their expression in grapevine tissues. Plant Physiology 111, 275–283. 8685267 10.1104/pp.111.1.275 PMC157835

[CIT0037] Davies C , Böttcher C, Nicholson EL, Burbidge CA, Boss PK. 2022. Timing of auxin treatment affects grape berry growth, ripening timing and the synchronicity of sugar accumulation. Australian Journal of Grape and Wine Research 28, 232–241.

[CIT0038] Deluc LG , Grimplet J, Wheatley MD, Tillett RL, Quilici DR, Osborne C, Schooley DA, Schlauch KA, Cushman JC, Cramer GR. 2007. Transcriptomic and metabolite analyses of Cabernet Sauvignon grape berry development. BMC Genomics 8, 429. 18034876 10.1186/1471-2164-8-429 PMC2220006

[CIT0039] Dong Y , Duan S, Xia Q, et al. 2023. Dual domestications and origin of traits in grapevine evolution. Science 379, 892–901. 36862793 10.1126/science.add8655

[CIT0040] Famiani F , Farinelli D, Frioni T, Palliotti A, Battistelli A, Moscatello S, Walker RP. 2016. Malate as substrate for catabolism and gluconeogenesis during ripening in the pericarp of different grape cultivars. Biologia Plantarum 60, 155–162.

[CIT0041] Fasoli M , Richter CL, Zenoni S, Bertini E, Vitulo N, Dal Santo S, Dokoozlian N, Pezzotti M, Tornielli GB. 2018. Timing and order of the molecular events marking the onset of berry ripening in grapevine. Plant Physiology 178, 1187–1206. 30224433 10.1104/pp.18.00559 PMC6236592

[CIT0042] Fischer TC , Mirbeth B, Rentsch J, Sutter C, Ring L, Flachowsky H, Habegger R, Hoffmann T, Hanke M-V, Schwab W. 2014. Premature and ectopic anthocyanin formation by silencing of anthocyanidin reductase in strawberry (*Fragaria* × *ananassa*). New Phytologist 201, 440–451. 24117941 10.1111/nph.12528

[CIT0043] Fortes AM , Teixeira RT, Agudelo-Romero P. 2015. Complex interplay of hormonal signals during grape berry ripening. Molecules 20, 9326–9343. 26007186 10.3390/molecules20059326 PMC6272489

[CIT0044] Fuentes S , Sullivan W, Tilbrook J, Tyerman S. 2010. A novel analysis of grapevine berry tissue demonstrates a variety-dependent correlation between tissue vitality and berry shrivel. Australian Journal of Grape and Wine Research 16, 327–336.

[CIT0045] Gambetta JM , Holzapfel BP, Stoll M, Friedel M. 2021. Sunburn in grapes: a review. Frontiers in Plant Science 11, 604691. 33488654 10.3389/fpls.2020.604691 PMC7819898

[CIT0046] Gouot JC , Smith JP, Holzapfel BP, Walker AR, Barril C. 2019. Grape berry flavonoids: a review of their biochemical responses to high and extreme high temperatures. Journal of Experimental Botany 70, 397–423. 30388247 10.1093/jxb/ery392

[CIT0047] Gouthu S , Deluc LG. 2015. Timing of ripening initiation in grape berries and its relationship to seed content and pericarp auxin levels. BMC Plant Biology 15, 46. 25848949 10.1186/s12870-015-0440-6 PMC4340107

[CIT0048] Greer DH , Rogiers SY. 2009. Water flux of *Vitis vinifera* L cv Shiraz bunches throughout development and in relation to late-season weight loss. American Journal of Enology and Viticulture 60, 155–163.

[CIT0049] Griesser M , Crespo Martinez S, Weidinger ML, Kandler W, Forneck A. 2017. Challenging the potassium deficiency hypothesis for induction of the ripening disorder berry shrivel in grapevine. Scientia Horticulturae 216, 141–147.

[CIT0050] Griesser M , Eder R, Besser S, Forneck A. 2012. Berry shrivel of grapes in Austria. Aspects of the physiological disorder with cultivar Zweigelt (*Vitis vinifera* L). Scientia Horticulturae 145, 87–93.

[CIT0051] Griesser M , Martinez SC, Eitle MW, Warth B, Andre CM, Schuhmacher R, Forneck A. 2018. The ripening disorder berry shrivel affects anthocyanin biosynthesis and sugar metabolism in Zweigelt grape berries. Planta 247, 471–481. 29075874 10.1007/s00425-017-2795-4 PMC5778156

[CIT0052] Griesser M , Savoi S, Supapvanich S, Dobrev P, Vankova R, Forneck A. 2020. Phytohormone profiles are strongly altered during induction and symptom development of the physiological ripening disorder berry shrivel in grapevine. Plant Molecular Biology 103, 141–157. 32072393 10.1007/s11103-020-00980-6 PMC7170833

[CIT0123] Griesser M , Savoi S. 2023. Differentially expressed genes in berries and rachis of berry shrivel grape clusters. [Dataset]. Zenodo. doi:10.5281/zenodo.10003017

[CIT0053] Hall GE , Bondada BR, Keller M. 2010. Loss of rachis cell viability is associated with ripening disorders in grapes. Journal of Experimental Botany 62, 1145–1153. 21071679 10.1093/jxb/erq355 PMC3022408

[CIT0054] Hayes MA , Davies C, Dry IB. 2007. Isolation, functional characterization, and expression analysis of grapevine (*Vitis vinifera* L) hexose transporters: differential roles in sink and source tissues. Journal of Experimental Botany 58, 1985–1997. 17452752 10.1093/jxb/erm061

[CIT0055] He F , Mu L, Yan GL, Liang NN, Pan QH, Wang J, Reeves MJ, Duan CQ. 2010. Biosynthesis of anthocyanins and their regulation in colored grapes. Molecules 15, 9057–9091. 21150825 10.3390/molecules15129057 PMC6259108

[CIT0056] Hernández-Montes E , Zhang Y, Chang B-M, Shcherbatyuk N, Keller M. 2021. Soft, sweet, and colorful: stratified sampling reveals sequence of events at the onset of grape ripening. American Journal of Enology and Viticulture 72, 137–151.

[CIT0057] Hewitt S , Hernández-Montes E, Dhingra A, Keller M. 2023. Impact of heat stress, water stress, and their combined effects on the metabolism and transcriptome of grape berries. Scientific Reports 13, 9907. 37336951 10.1038/s41598-023-36160-x PMC10279648

[CIT0058] Hifny HAA , Alleweldt G. 1972. Untersuchungen zur Stiellähme der Reben I Die Symptomatologie der Krankheit. Vitis 10, 298–313.

[CIT0059] Hoff RT , Bondada BR, Keller M. 2021. Onset and progression of the berry shrivel ripening disorder in grapes. Australian Journal of Grape and Wine Research 27, 280–289.

[CIT0060] Holzapfel BP , Coombe BG. 1998. Interaction of perfused chemicals as inducers and reducers of bunchstem necrosis in grapevine bunches and the effects on the bunchstem concentrations of ammonium ion and abscisic acid. Australian Journal of Grape and Wine Research 4, 59–66.

[CIT0061] Hughes E , Reynolds A, Bondada B. 2008. Bunch stem necrosis. Wine East 35, 18–25.

[CIT0062] Jackson DI , Coombe BG. 1988. Early bunchstem necrosis in grapes—a cause of poor fruit set. Vitis 27, 57–61.

[CIT0063] Jensen FL. 1970. Effects of post-bloom gibberellin application on berry shrivel and berry weight on seeded *Vitis vinifera* table grapes. MS thesis, Viticulture and Enology, University of California, Davis.

[CIT0064] Keller M , Koblet W. 1995. Stress-induced development of inflorescence necrosis and bunch-stem necrosis in *Vitis vinifera* L in response to environmental and nutritional effects. Vitis 34, 145–150.

[CIT0065] Keller M , Scheele-Baldinger R, Ferguson JC, Tarara JM, Mills LJ. 2022. Inflorescence temperature influences fruit set, phenology, and sink strength of Cabernet Sauvignon grape berries. Frontiers in Plant Science 13, 864892. 36046582 10.3389/fpls.2022.864892 PMC9420974

[CIT0066] Keller M , Shrestha PM. 2014. Solute accumulation differs in the vacuoles and apoplast of ripening grape berries. Planta 239, 633–642. 24310282 10.1007/s00425-013-2004-z

[CIT0067] Keller M , Shrestha PM, Hall GE, Bondada BR, Davenport JR. 2016. Arrested sugar accumulation and altered organic acid metabolism in grape berries affected by berry shrivel syndrome. American Journal of Enology and Viticulture 67, 398.

[CIT0068] Keller M , Smith JP, Bondada BR. 2006. Ripening grape berries remain hydraulically connected to the shoot. Journal of Experimental Botany 57, 2577–2587. 16868045 10.1093/jxb/erl020

[CIT0069] Keller M , Zhang Y, Shrestha PM, Biondi M, Bondada BR. 2015. Sugar demand of ripening grape berries leads to recycling of surplus phloem water via the xylem. Plant, Cell & Environment 38, 1048–1059. 10.1111/pce.12465 25293537

[CIT0070] Knoll M , Achleitner D, Redl H. 2010. Sugar accumulation in ‘Zweigelt’ grapes as affected by ‘Traubenwelke’. Vitis 49, 101–106.

[CIT0071] Krasnow M , Matthews M, Shackel K. 2008. Evidence for substantial maintenance of membrane integrity and cell viability in normally developing grape (*Vitis vinifera* L) berries throughout development. Journal of Experimental Botany 59, 849–859. 18272917 10.1093/jxb/erm372

[CIT0072] Krasnow M , Weis N, Smith RJ, Benz MJ, Matthews M, Shackel K. 2009. Inception, progression, and compositional consequences of a berry shrivel disorder. American Journal of Enology and Viticulture 60, 24–34.

[CIT0073] Krasnow MN , Matthews MA, Smith RJ, Benz J, Weber E, Shackel KA. 2010. Distinctive symptoms differentiate four common types of berry shrivel disorder in grape. California Agriculture 64, 155–159.

[CIT0074] Kuhn N , Guan L, Dai ZW, Wu BH, Lauvergeat V, Gomès E, Li SH, Godoy F, Arce-Johnson P, Delrot S. 2014. Berry ripening: recently heard through the grapevine. Journal of Experimental Botany 65, 4543–4559. 24285825 10.1093/jxb/ert395

[CIT0075] Lecourieux D , Kappel C, Claverol S, et al. 2020. Proteomic and metabolomic profiling underlines the stage- and time-dependent effects of high temperature on grape berry metabolism. Journal of Integrative Plant Biology 62, 1132–1158. 31829525 10.1111/jipb.12894

[CIT0076] Lecourieux F , Kappel C, Lecourieux D, Serrano A, Torres E, Arce-Johnson P, Delrot S. 2013. An update on sugar transport and signalling in grapevine. Journal of Experimental Botany 65, 821–832. 24323501 10.1093/jxb/ert394

[CIT0077] Li Y-M , Forney C, Bondada B, Leng F, Xie Z-S. 2021. The molecular regulation of carbon sink strength in grapevine (*Vitis vinifera* L). Frontiers in Plant Science 11, 606918. 33505415 10.3389/fpls.2020.606918 PMC7829256

[CIT0078] Lin J , Massonnet M, Cantu D. 2019. The genetic basis of grape and wine aroma. Horticulture Research 6, 81. 31645942 10.1038/s41438-019-0163-1 PMC6804543

[CIT0079] Ma B , Yuan Y, Gao M, Li C, Ogutu C, Li M, Ma F. 2018. Determination of predominant organic acid components in *Malus* species: correlation with apple domestication. Metabolites 8, 74. 30384454 10.3390/metabo8040074 PMC6316603

[CIT0080] Müller K , Keller M, Stoll M, Friedel M. 2023. Wind speed, sun exposure and water status alter sunburn susceptibility of grape berries. Frontiers in Plant Science 14, 1145274. 37051085 10.3389/fpls.2023.1145274 PMC10083509

[CIT0081] Nonis A , Ruperti B, Pierasco A, Canaguier A, Adam-Blondon AF, Di Gaspero G, Vizzotto G. 2008. Neutral invertases in grapevine and comparative analysis with Arabidopsis, poplar and rice. Planta 229, 129–142. 18800225 10.1007/s00425-008-0815-0

[CIT0082] Osterwalder A. 1937. Vorzeitiges Welken von Trauben, eine noch wenig bekannte Art Lahmstiehler. Schweizer Zeitschrift Obst-Weinbau 46, 428–432.

[CIT0083] Palumbo MC , Zenoni S, Fasoli M, Massonnet M, Farina L, Castiglione F, Pezzotti M, Paci P. 2014. Integrated network analysis identifies fight-club nodes as a class of hubs encompassing key putative switch genes that induce major transcriptome reprogramming during grapevine development. The Plant Cell 26, 4617–4635. 25490918 10.1105/tpc.114.133710 PMC4311215

[CIT0084] Pilati S , Bagagli G, Sonego P, et al. 2017. Abscisic acid is a major regulator of grape berry ripening onset: new insights into ABA signaling network. Frontiers in Plant Science 8, 1093. 28680438 10.3389/fpls.2017.01093 PMC5479058

[CIT0085] Pimentel D , Amaro R, Erban A, Mauri N, Soares F, Rego C, Martínez-Zapater JM, Mithöfer A, Kopka J, Fortes AM. 2021. Transcriptional, hormonal, and metabolic changes in susceptible grape berries under powdery mildew infection. Journal of Experimental Botany 72, 6544–6569. 34106234 10.1093/jxb/erab258

[CIT0086] Qin L , Xie H, Xiang N, Wang M, Han S, Pan M, Guo X, Zhang W. 2022. Dynamic changes in anthocyanin accumulation and cellular antioxidant activities in two varieties of grape berries during fruit maturation under different climates. Molecules 27, 384. 35056697 10.3390/molecules27020384 PMC8782009

[CIT0087] Raifer B , Haas F, Cassar A. 2015. Influence of leaf canopy height on the occurrence of berry shrivel. Vitis 53, 117–123.

[CIT0088] Ren R , Yue X, Li J, Xie S, Guo S, Zhang Z. 2020. Coexpression of sucrose synthase and the SWEET transporter, which are associated with sugar hydrolysis and transport, respectively, increases the hexose content in *Vitis vinifera* L grape berries. Frontiers in Plant Science 11, 321. 32457764 10.3389/fpls.2020.00321 PMC7221319

[CIT0089] Ren Y , Liao S, Xu Y. 2023. An update on sugar allocation and accumulation in fruits. Plant Physiology 193, 888–899. 37224524 10.1093/plphys/kiad294

[CIT0090] Rienth M , Vigneron N, Walker RP, Castellarin SD, Sweetman C, Burbidge CA, Bonghi C, Famiani F, Darriet P. 2021. Modifications of grapevine berry composition induced by main viral and fungal pathogens in a climate change scenario. Frontiers in Plant Science 12, 717223. 34956249 10.3389/fpls.2021.717223 PMC8693719

[CIT0091] Robinson AL , Boss PK, Solomon PS, Trengove RD, Heymann H, Ebeler SE. 2014. Origins of grape and wine aroma. Part 1. Chemical components and viticultural impacts. American Journal of Enology and Viticulture 65, 1–24.

[CIT0092] Rogiers SY , Greer DH, Hatfield JM, Orchard BA, Keller M. 2006. Solute transport into Shiraz berries during development and late-ripening shrinkage. American Journal of Enology and Viticulture 57, 73–80.

[CIT0093] Rosa WVL , Nielson U. 1956. Effect of delay in harvesting on the composition of grapes. American Journal of Enology and Viticulture 7, 105–111.

[CIT0094] Rustioni L , Altomare A, Shanshiashvili G, Greco F, Buccolieri R, Blanco I, Cola G, Fracassetti D. 2023. Microclimate of grape bunch and sunburn of white grape berries: effect on wine quality. Foods 12, 621. 36766149 10.3390/foods12030621 PMC9914167

[CIT0095] Rustioni L , Rocchi L, Guffanti E, Cola G, Failla O. 2014. Characterization of grape (*Vitis vinifera* L) berry sunburn symptoms by reflectance. Journal of Agricultural and Food Chemistry 62, 3043–3046. 24650184 10.1021/jf405772f

[CIT0096] Savoi S , Herrera JC, Forneck A, Griesser M. 2019. Transcriptomics of the grape berry shrivel ripening disorder. Plant Molecular Biology 100, 285–301. 30941542 10.1007/s11103-019-00859-1 PMC6542784

[CIT0097] Savoi S , Supapvanich S, Hildebrand H, Stralis-Pavese N, Forneck A, Kreil DP, Griesser M. 2022. Expression analyses in the rachis hint towards major cell wall modifications in grape clusters showing berry shrivel symptoms. Plants 11, 2159. 36015462 10.3390/plants11162159 PMC9413262

[CIT0098] Savoi S , Torregrosa L, Romieu C. 2021. Transcripts switched off at the stop of phloem unloading highlight the energy efficiency of sugar import in the ripening *V. vinifera* fruit. Horticulture Research 8, 193. 34465746 10.1038/s41438-021-00628-6 PMC8408237

[CIT0099] Savoi S , Wong DCJ, Degu A, Herrera JC, Bucchetti B, Peterlunger E, Fait A, Mattivi F, Castellarin SD. 2017. Multi-omics and integrated network analyses reveal new insights into the systems relationships between metabolites, structural genes, and transcriptional regulators in developing grape berries (*Vitis vinifera* L) exposed to water deficit. Frontiers in Plant Science 8, 1124. 28740499 10.3389/fpls.2017.01124 PMC5502274

[CIT0100] Shahood R , Torregrosa L, Savoi S, Romieu C. 2020. First quantitative assessment of growth, sugar accumulation and malate breakdown in a single ripening berry. OENO One 54, 1077–1092.

[CIT0101] Shi Y , Li BJ, Grierson D, Chen KS. 2023. Insights into cell wall changes during fruit softening from transgenic and naturally occurring mutants. Plant Physiology 192, 1671–1683. 36823689 10.1093/plphys/kiad128 PMC10315278

[CIT0102] Siebert TE , Barter SR, de Barros Lopes MA, Herderich MJ, Francis IL. 2018. Investigation of ‘stone fruit’ aroma in Chardonnay, Viognier and botrytis Semillon wines. Food Chemistry 256, 286–296. 29606450 10.1016/j.foodchem.2018.02.115

[CIT0103] Stein O , Granot D. 2019. An overview of sucrose synthases in plants. Frontiers in Plant Science 10, 95. 30800137 10.3389/fpls.2019.00095 PMC6375876

[CIT0104] Stellwaag-Kittler F. 1983. Aeussere Symptomatik der Stiellähme an Trauben. Mitteilungen Klosterneuburg 33, 94–99.

[CIT0105] Šuklje K , Zhang X, Antalick G, Clark AC, Deloire A, Schmidtke LM. 2016. Berry shriveling significantly alters Shiraz (*Vitis vinifera* L) grape and wine chemical composition. Journal of Agricultural and Food Chemistry 64, 870–880. 26761394 10.1021/acs.jafc.5b05158

[CIT0106] Sun L , Zhang M, Ren J, Qi J, Zhang G, Leng P. 2010. Reciprocity between abscisic acid and ethylene at the onset of berry ripening and after harvest. BMC Plant Biology 10, 257. 21092180 10.1186/1471-2229-10-257 PMC3095336

[CIT0107] Sweetman C , Deluc LG, Cramer GR, Ford CM, Soole KL. 2009. Regulation of malate metabolism in grape berry and other developing fruits. Phytochemistry 70, 1329–1344. 19762054 10.1016/j.phytochem.2009.08.006

[CIT0108] Terrier N , Glissant D, Grimplet J, et al. 2005. Isogene specific oligo arrays reveal multifaceted changes in gene expression during grape berry (*Vitis vinifera* L) development. Planta 222, 832–847. 16151847 10.1007/s00425-005-0017-y

[CIT0109] Tilbrook J , Tyerman SD. 2008. Cell death in grape berries: varietal differences linked to xylem pressure and berry weight loss. Functional Plant Biology 35, 173–184. 32688771 10.1071/FP07278

[CIT0110] Ugalde JM , Lamig L, Herrera-Vásquez A, Fuchs P, Homagk M, Kopriva S, Müller-Schüssele SJ, Holuigue L, Meyer AJ. 2021. A dual role for glutathione transferase U7 in plant growth and protection from methyl viologen-induced oxidative stress. Plant Physiology 187, 2451–2468. 34599589 10.1093/plphys/kiab444 PMC8644736

[CIT0111] Ul Haq S , Khan A, Ali M, Khattak AM, Gai WX, Zhang HX, Wei AM, Gong ZH. 2019. Heat shock proteins: dynamic biomolecules to counter plant biotic and abiotic stresses. International Journal of Molecular Sciences 20, 5321. 31731530 10.3390/ijms20215321 PMC6862505

[CIT0112] Walker RP , Bonghi C, Varotto S, et al. 2021a. Sucrose metabolism and transport in grapevines, with emphasis on berries and leaves, and insights gained from a cross-species comparison. International Journal of Molecular Sciences 22, 7794. 34360556 10.3390/ijms22157794 PMC8345980

[CIT0113] Walker RP , Chen ZH, Famiani F. 2021b. Gluconeogenesis in plants: a key interface between organic acid/amino acid/lipid and sugar metabolism. Molecules 26, 5129. 34500562 10.3390/molecules26175129 PMC8434439

[CIT0114] Winkler AJ , Williams WO. 1935. Effect of seed development on the growth of grapes. Proceedings of the American Society for Horticultural Sciences 33, 430–434.

[CIT0115] Xiao Z , Rogiers SY, Sadras VO, Tyerman SD. 2018. Hypoxia in grape berries: the role of seed respiration and lenticels on the berry pedicel and the possible link to cell death. Journal of Experimental Botany 69, 2071–2083. 29415235 10.1093/jxb/ery039 PMC6018838

[CIT0116] Xu Q , Yin S, Ma Y, et al. 2020. Carbon export from leaves is controlled via ubiquitination and phosphorylation of sucrose transporter SUC2. Proceedings of the National Academy of Sciences, USA 117, 6223–6230. 10.1073/pnas.1912754117 PMC7084081 32123097

[CIT0117] Ye C , Zheng S, Jiang D, Lu J, Huang Z, Liu Z, Zhou H, Zhuang C, Li J. 2021. Initiation and execution of programmed cell death and regulation of reactive oxygen species in plants. International Journal of Molecular Sciences 22, 12942. 34884747 10.3390/ijms222312942 PMC8657872

[CIT0118] Zhang XY , Wang XL, Wang XF, Xia GH, Pan QH, Fan RC, Wu FQ, Yu XC, Zhang DP. 2006. A shift of phloem unloading from symplasmic to apoplasmic pathway is involved in developmental onset of ripening in grape berry. Plant Physiology 142, 220–232. 16861573 10.1104/pp.106.081430 PMC1557625

[CIT0119] Zhang Y , Keller M. 2015. Grape berry transpiration is determined by vapor pressure deficit, cuticular conductance, and berry size. American Journal of Enology and Viticulture 66, 454–462.

[CIT0120] Zhang Y , Keller M. 2017. Discharge of surplus phloem water may be required for normal grape ripening. Journal of Experimental Botany 68, 585–595. 28082510 10.1093/jxb/erw476 PMC5444433

[CIT0121] Zhang Z , Zou L, Ren C, Ren F, Wang Y, Fan P, Li S, Liang Z. 2019. VvSWEET10 mediates sugar accumulation in grapes. Genes 10, 255. 30925768 10.3390/genes10040255 PMC6523336

[CIT0122] Zufferey V , Spring J-L, Voinesco F, Viret O, Gindro K. 2015. Physiological and histological approaches to study berry shrivel in grapes. OENO One 49, 113–125.

